# Glenosphere Tilt and Size Predict Shoulder Kinematics During the Hand‐to‐Back Motion After Reverse Shoulder Arthroplasty

**DOI:** 10.1002/jor.70072

**Published:** 2025-09-30

**Authors:** Ajinkya A. Rai, Clarissa M. LeVasseur, Gillian E. Kane, Maria A. Munsch, Christopher J. Como, Alexandra S. Gabrielli, Jonathan D. Hughes, William J. Anderst, Albert Lin

**Affiliations:** ^1^ Biodynamics Laboratory University of Pittsburgh Pittsburgh PA; ^2^ Department of Orthopaedic Surgery University of Pittsburgh Pittsburgh PA

**Keywords:** arthroplasty, kinematics, outcomes, reverse, shoulder

## Abstract

Internal rotation (IR) is not reliably improved after reverse shoulder arthroplasty (RSA). This study aimed to identify surgical parameters that predict kinematics of the hand‐to‐back motion (H2B) after RSA and to identify associations between kinematics and clinical outcomes after RSA. We hypothesized that less humeral retroversion, more lateralization and a larger glenosphere would predict kinematics associated with favorable outcomes post‐RSA. Thirty‐five patients performed H2B while synchronized biplane radiographs were collected. Digitally reconstructed radiographs, constructed from patient‐specific bone plus implant models, were matched to the biplane radiographs to determine kinematics. The total contribution to motion, the end position, peak angles, and range of motion (ROM) were found for all glenohumeral and scapular rotations. The path of the center of the humeral insert on the glenosphere was calculated. Patient‐reported outcomes, clinical ROM, and strength were measured. Associations were determined between intraoperative variables and kinematics as well as between kinematics and outcomes. The results demonstrated that glenosphere tilt predicted glenohumeral and scapular kinematics; these kinematics were associated with IR ROM, strength, and more favorable patient‐reported outcomes. A larger glenosphere predicted a center of contact that was associated with more strength in IR. All components of scapular rotation were associated with favorable outcomes, suggesting rehabilitation focusing on scapular motion may improve outcomes post‐RSA. Glenosphere tilt and size predicted kinematics that were associated with range of motion, strength, and patient‐reported outcomes.

## 1 Introduction

Internal rotation (IR) is evaluated as patients attempt to reach their affected‐side hand as far up as possible on their spine, also known as the “hand‐to‐back” motion (H2B) [1], which mimics many activities of daily living such as washing lower back, unstrapping bra, etc [2, 3, 4]. At follow‐up after reverse shoulder arthroplasty (RSA), only 36.4% of patients can effectively wash their back and generally do not recover Preoperative range of motion (ROM) [5].

Much research has been conducted to identify how surgical decisions during RSA influence the ability to effectively perform IR, especially in the context of H2B. Glenoid lateralization [6, 7, 8], glenosphere size [9, 10, 11] and tilt [12], neckshaft angle [13], and humeral anteversion [13] have all previously been associated with postoperative ROM and strength, however, the findings were inconsistent [14, 15]. Thus, the ideal implant configuration to optimize IR after RSA remains controversial [16].

Little research has been done to reveal how surgical decisions influence in vivo kinematics after RSA and which kinematics are associated with favorable clinical outcomes. Como et al. reported that patients with neck‐shaft angles of 145° had more ROM during H2B when compared to patients with 135° neck‐shaft implants [17] and also reported that during H2B more glenoid lateralization led to less IR, and more abduction and IR were associated with favorable clinical outcomes [17]. However, that study utilized traditional motion capture and markers placed on skin, which are known to be inaccurate for measuring glenohumeral motion [18], resulting on average errors as high as 14° [19]. Biplane radiography is an alternative technique that can measure skeletal motion without soft tissue artifact [20]. Using biplanar technology, Sulkar, et al. found that scapular upward rotation and tilt may be favorable kinematics for H2B performance after RSA [21]. However, while previous in vivo analysis has studied the hand‐to‐head motion [22], no studies have identified any links between surgical parameters, kinematics, and clinical outcomes after RSA during H2B.

**Figure 1:**
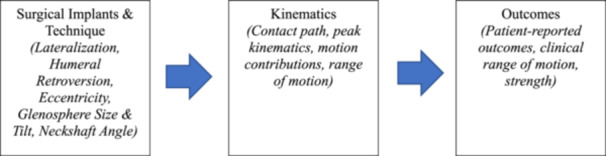
The flow chart of our study. We aimed to discover if surgical decisions influenced any kinematics of the H2B motion, and then we sought to discover any associations between kinematics and patient outcomes at testing.

The first aim of this study was to identify RSA implant placement and design features that may influence in vivo kinematics and the contact path of the humeral implant on the glenosphere during the H2B motion after RSA. The second aim was to identify which kinematic parameters are associated with clinical outcomes (Figure 1). We hypothesized that inferior glenosphere tilt, less humeral retroversion, and more lateralization would be associated with scapular upward rotation and tilt, as well as contact path patterns, during the H2B motion, and that associated kinematics would predict favorable patient‐reported outcomes (PROs).

## 2 Methods

All participants provided written informed consent before beginning this IRB‐approved study. Inclusion criteria were: at least 18 years of age at testing, patients who received RSA and completed the postoperative healing period within the previous 1–5 years for atraumatic rotator cuff arthropathy, irreparable cuff tear, or arthritis, and patients must have completed a supervised postoperative physical therapy and rehabilitative program (Table 1). RSA was performed by a single fellowship‐trained shoulder surgeon using a standard 135° inlay humeral implant (Arthrex; Naples, Florida, USA) or a 145° onlay humeral implant (Tornier, Stryker; Kalamazoo, MI, USA). The surgeon switched from using one implant on all patients to using the other implant on all patients in the latter half of the 5‐year window of patients. The glenoid was placed in neutral alignment for all patients. Subject screening was initiated at least 1 year after RSA, but before any kinematics or outcome measures were recorded. Surgical notes were reviewed to screen for patients who could provide a range of lateralization, glenosphere size, eccentricity, and neck‐shaft angle. Glenoid tilt and humeral retroversion were calculated based on 3D bone models based off anatomical coordinate systems, as described below. At testing, no patients had revision procedures.

**Table 1:** Patient Demographics, Preoperative Diagnosis, and Rotator Cuff Status.

Subject	Age (years)	Sex	BMI	Time from surgery (years)	Shoulder	Hand dominance	Pre‐Op diagnosis	Rotator Cuff Status (I‐Intact, T‐Torn, A‐Attenuated)			
								Supraspinatus	Infraspinatus	Teres minor	Subscapularis
1	64.19	F	46.2	3.24	L	R	Osteoarthritis	A	I	I	I
2	69.80	M	26.6	1.28	R	R	Massive cuff tear	T	T	I	I
3	63.68	M	31.5	3.31	R	R	Rotator cuff arthropathy	T	T	I	T
4	68.99	F	33.2	3.83	R	R	Osteoarthritis	T	I	I	A
5	67.05	F	27.1	4.52	R	R	Rotator cuff arthropathy	T	T	I	A
6	79.97	F	23.3	3.63	R		Osteoarthritis				
7	68.77	F	35	1.52	L	R	Rotator cuff arthropathy	T	I	I	T
8	62.85	M	27.9	1.85	R	R	Rotator cuff arthropathy	T	T	I	A
9	70.80	M	27.6	3.76	R	R	Rotator cuff arthropathy	A	I	I	I
10	70.84	M	23.7	1.75	R	R	Massive cuff tear	T	T	I	A
11	74.12	M	27.2	1.05	R	R	Rotator cuff arthropathy	T	T	I	I
12	56.28	M	33.2	1.08	L	L	Rotator cuff arthropathy	T	T	I	I
13	87.12	F	30.3	1.04	R	R	Rotator cuff arthropathy	T	T	I	T
14	76.33	M	31.7	1.10	L	R	Rotator cuff arthropathy	I	I	I	I
15	73.65	M	22.7	3.47	R	R	Rotator cuff arthropathy	T	T	I	I
16	76.82	F	27.5	3.21	L	R	Rotator cuff arthropathy	T	A	I	I
17	69.69	M	30.9	0.95	L	R	Rotator cuff arthropathy	A	I	I	A
18	70.61	F	32.2	2.06	R	R	Rotator cuff arthropathy	T	T	I	A
19	88.43	F	27.0	3.19	R	R	Rotator cuff arthropathy	T	T	I	I
20	81.02	F	25.2	4.41	L	R	Rotator cuff arthropathy	T	T	I	I
21	70.39	F	29.7	2.68	L	R	Chronic posttraumatic	A	A	I	I
22	67.39	M	19.6	3.35	L	R	Rotator cuff arthropathy	T	I	I	T
23	85.29	F	32.9	2.60	R	R	Massive cuff tear	T	T	I	I
24	70.97	M	31.8	2.02	L	R	Osteoarthritis	I	I	I	I
25	69.52	M	33.1	2.24	L	R	Rotator cuff arthropathy	T	T	I	A
26	78.80	F	25.1	1.11	R	R	Acute posttraumatic	I	I	I	I
27	63.26	M	46.2	1.02	R	R	Osteoarthritis	I	I	I	I
28	78.47	M	28.0	1.02	L	L	Rotator cuff arthropathy	T	T	I	A
29	70.64	F	28.8	1.12	R	R	Rotator cuff arthropathy	T	T	I	A
30	76.25	F	22.2	1.47	L	R	Rotator cuff arthropathy	A	A	I	I
31	74.91	F	26.4	1.65	R	R	Rotator cuff arthropathy	T	T	I	I
32	70.62	M	26.7	2.05	R	R	Rotator cuff arthropathy	T	T	I	I
33	80.90	F	34.5	1.73	R	R	Chronic posttraumatic	I	I	I	I
34	66.26	M	26.1	1.55	R	R	Osteoarthritis	I	I	I	I
35	83.21	F	26.7	2.44	L	R	Osteoarthritis	I	I	I	I

All patients remained immobilized in a sling for 6 weeks after surgery. After 6 weeks, the sling was discontinued and formal therapy was initiated by a licensed physical therapist. The standardized therapy protocol for all patients was initiated with gentle passive and active range of motion, followed by strengthening beginning at the 12‐week mark. Depending on clinical progress and physical therapist evaluation, patients were graduated from physical therapy at the 12‐week mark, at which point patients were instructed to continue home exercises until the 1‐year follow‐up appointment. At the 1‐year follow‐up, patients were cleared to return to full activity after a clinical evaluation by the operating surgeon.

In vivo kinematics were recorded using conventional motion capture and biplane radiography simultaneously. Reflective markers were placed on the styloid process of the radius and ulna, medial and lateral epicondyles on the humerus, the bicep, acromioclavicular joint bilaterally, the manubrium, xiphoid process, C7, T10, and the inferior angle of the right scapula. Participants sat upright within a custom biplane radiography system and were instructed to look straight ahead with their hand on the lap for a single static trial. Participants were then instructed to move their hand from their lap to the back of their spine following an example demonstration by a research team member. Participants were encouraged to reach as far up as possible while maintaining the upright posture. No other specific instructions were given on how to perform the movement. During the motion, synchronized biplane radiographs of the shoulder were captured at 50 images per second for 2 s (imaging parameters: 90 kV, 50 mA, 2 ms pulse width) while conventional motion capture was synchronously collected with the biplane radiographs at 100 Hz using a 12‐camera system (Vicon motion systems, Oxford UK). Three separate movement trials were collected for each participant. The maximum radiation exposure during all dynamic trials using the biplane radiography system was estimated to be 1.87 mSv (estimated using PCXMC, STUK – Radiation and Nuclear Safety Authority, Helsinki, Finland).

**Figure 2:**
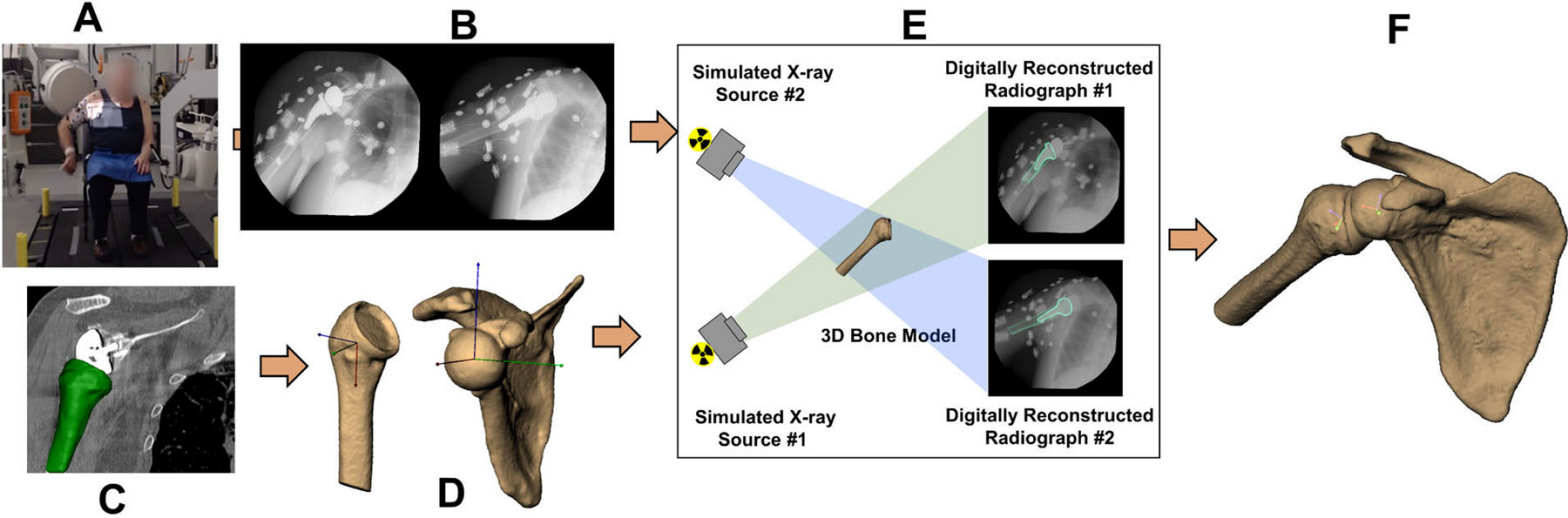
Biplane radiography data collection and processing. (A) Participants performed three H2B movements while (B) synchronized biplane radiographs were collected at 50 images per second (90 kV, 50 mA, 2 ms pulse width). (C) Scapula and humerus computed tomography (CT) scans were collected (0.47 × 0.47 × 0.625 mm) and (D) used to create three‐dimensional (3D) bone models. (E) 3D shoulder kinematics were determined using a validated CT model‐based tracking process. (F) Six degree‐of‐freedom kinematics were calculated throughout the full range of motion.

Computed tomography (CT) scans with metal artifact reduction (0.47 × 0.47 × 0.625‐mm voxels) of the affected shoulder and distal humerus were acquired from each participant (GE Lightspeed, Waukesha, WI) and were resliced to generate cubic voxels (Figure 2). Implants and bone tissue of the humerus and scapula were segmented from the CT for each participant using a combination of commercial software (Materalise, NV, Leuven, Belgium) and manual segmentation. Markers were interactively placed on the trigonum spinae, angulus inferior, and angulus acromialis of the three‐dimensional (3D) scapula, and on the glenosphere as well as the center of the humeral tray and medial and lateral epicondyles on the distal humerus to create subject‐specific coordinate systems for both the humerus and scapula according to International Society of Biomechanics standards [23]. Humeral retroversion and glenosphere tilt were calculated on the 3D bone models based on the anatomical coordinate systems established in each bone.

In vivo scapular and humeral motion were determined by matching digitally reconstructed radiographs, created from subject‐specific segmented bone tissue of the humerus and scapula with their respective implants, to biplane radiographs with sub‐millimeter accuracy [20] (Figure 2). Glenohumeral (GH) kinematics were calculated in Matlab following a previously described algorithm that uses spherical coordinates [24, 25], rather than Euler angles, as this method is easier for clinicians to understand and it mimics practical measurement methods commonly employed by physicians, therapists, etc [24, 25]. to describe the GH plane of elevation, GH elevation, and internal/external rotation. Scapular upward rotation was calculated relative to the thorax, with thorax motion defined by the reflective markers placed on the manubrium, xiphoid process, C7, T10, and opposite acromion process. Each kinematics data set was time‐normalized to the percent of the movement such that 0% corresponded to the hand beginning to move from the lap and 100% corresponded to the hand stopping on the back of the spine as determined by the velocity of the reflective markers placed on the wrist. The average within‐subject variability in time to complete the motion was 0.14 s (< 7% of the total movement time). The three trials were staggered in terms of beginning the imaging capture so that the overlapping duration of three captures encompassed the entirety of beginning the motion through coming to a rest after the movement. All six scapular and GH rotations (scapular upward rotation, protraction, and tilt, and GH abduction, plane of elevation, and internal/external rotation) were averaged at corresponding percentages of the movement across the three movement repetitions for each participant. Peak GH abduction and adduction, anterior and posterior plane of elevation, scapular upward rotation, and external rotation, as well as total GH ab/adduction, plane of elevation, scapular upward rotation, and internal/external rotation were found from each subject's average kinematics curves. The average end position abduction, plane of elevation, internal/external rotation, and upward rotation were also calculated.

A 3D model of the humeral liner, provided by the manufacturers, was fit into the CT‐based humeral tray, and the humerus kinematics were used to drive the humeral tray kinematics. The movement of the geometric centroid of the triangulated surface of the humeral liner relative to the glenosphere was calculated for every frame of motion. The path of the liner center on the glenosphere in the superior/inferior (SI) and anterior/posterior (AP) directions was averaged across corresponding time points of the three movement cycles to establish an average path for the center of contact during the movement.

American Shoulder and Elbow Surgeons Standardized Shoulder Assessment Forms (ASES), Disabilities of the Arm, Shoulder, and Hand (DASH), and Constant‐Murley Shoulder Outcome (CMS) scores were collected at testing. Active clinical IR ROM was quantified by instructing the participants to place their arm as high up their back as they could and recording the spinal location of their thumb at the end position, then passive ROM was measured by the furthest the arm could go without resistance under assistance from the clinician. Vertebral levels were then numerically reclassified such that 1 corresponding to the hand reaching the buttock, 2 denoting the sacrum, 3 denoting lower lumbar (L4/L5), 4 denoting upper lumbar vertebra (L3‐L1) and lower thoracic vertebra (T12‐T10), and 5 denoting interscapular level. A Biodex machine set to 30° per second was used to measure isokinetic torque throughout the full ROM of IR and ER with the arm at 90° of abduction in a seated position. Peak torque and total work done during both IR and ER were calculated from the torque/angle curves and normalized to bodyweight.

Multiple linear regression using forward selection was used to test if implant characteristics and surgical techniques (lateralization, glenosphere size and tilt, neck‐shaft angle, and humeral retroversion; eccentricity was not assessed due to lack of variability among patients) predicted kinematics (peak angles, end position, peak center of contact path locations) (SPSS 29.0 software) with a probability of F < 0.05 to enter. The unstandardized beta coefficients were reported so that the reader can understand the change in the dependent variable for a one‐unit change in each independent variable. Associations between kinematics and clinical outcomes (patient‐reported outcomes (PROs), clinical ROM, and strength) were evaluated with Pearson's correlations. For our secondary analysis, associations between surgical parameters and clinical outcomes were evaluated with Pearson's correlations. The strength of the correlation was classified as poor (*r* < 0.3), fair (*r* = 0.3 to 0.5), moderate (*r* = 0.5–0.8), or strong (*r* ≥ 0.8) [26]. Considering that no in vivo data exist describing the relationship between kinematics and surgical techniques nor implant characteristics, nor between kinematics and clinical outcomes after RSA, we did not want to overlook potential important associations; therefore, significance was set at *p* < 0.05 for all analyses.

## 3 Results

### 3.1 Demographics

**Figure 3:**
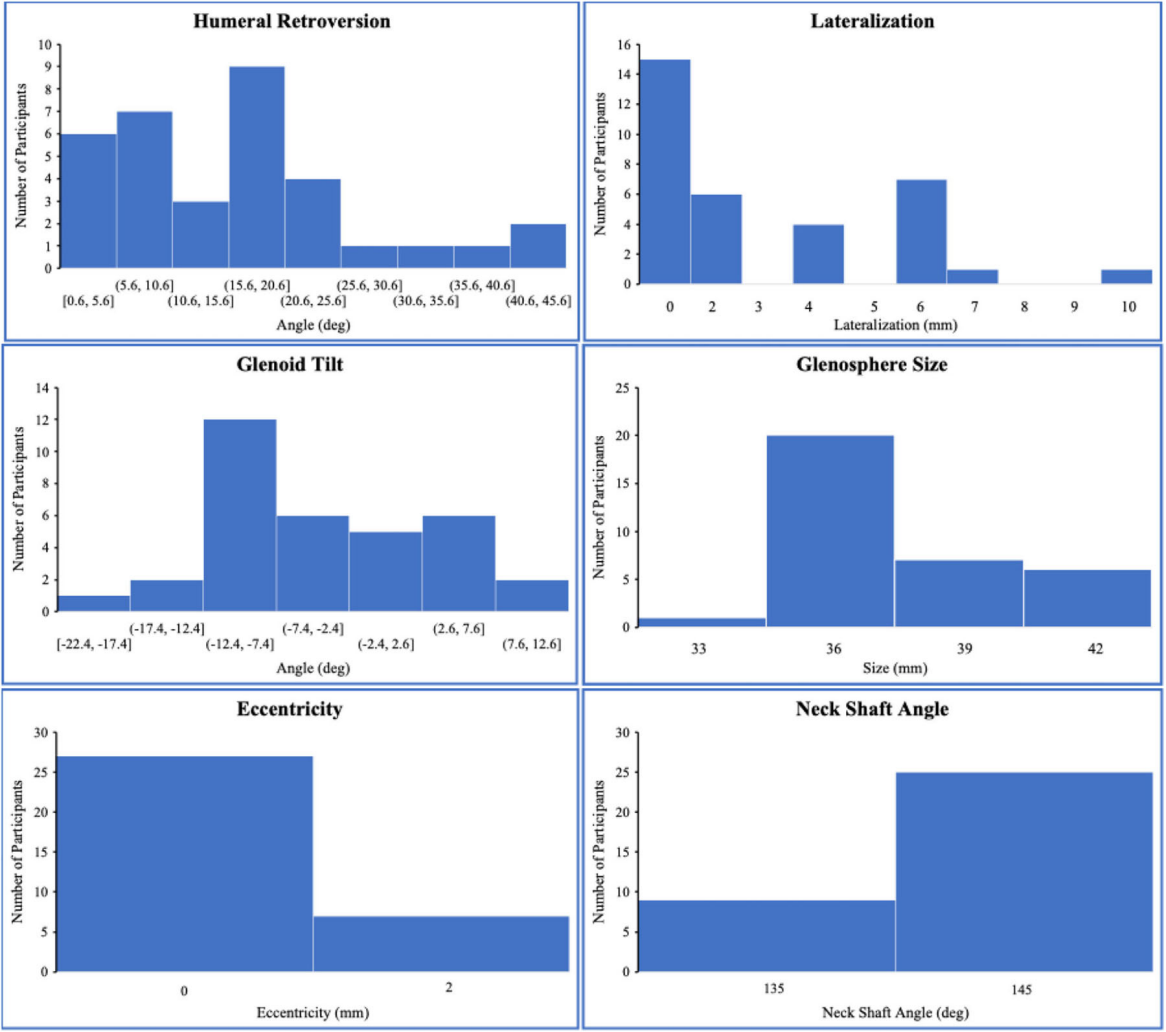
Distribution of surgical parameters. Angles were reported in degrees (°); lateralization, eccentricity, and glenosphere size were reported in millimeters (mm).

This study included 35 patients who received RSA (17 M, 18 F, 72.8 ± 7.3 years) an average of 2.2 ± 1.1 years before study participation (Table 1). Thirty‐four patients were analyzed; one patient was excluded because their RSA indication was acute posttraumatic tear. A total of 97 trials of the H2B motion were included in this analysis; five trials were excluded due to the subject moving out of the imaging volume. The conventional motion capture kinematics for this cohort have been previously published [17]. The distribution of implant parameters and placement is shown in Figure 3.

### 3.2 Kinematics

**Figure 4:**
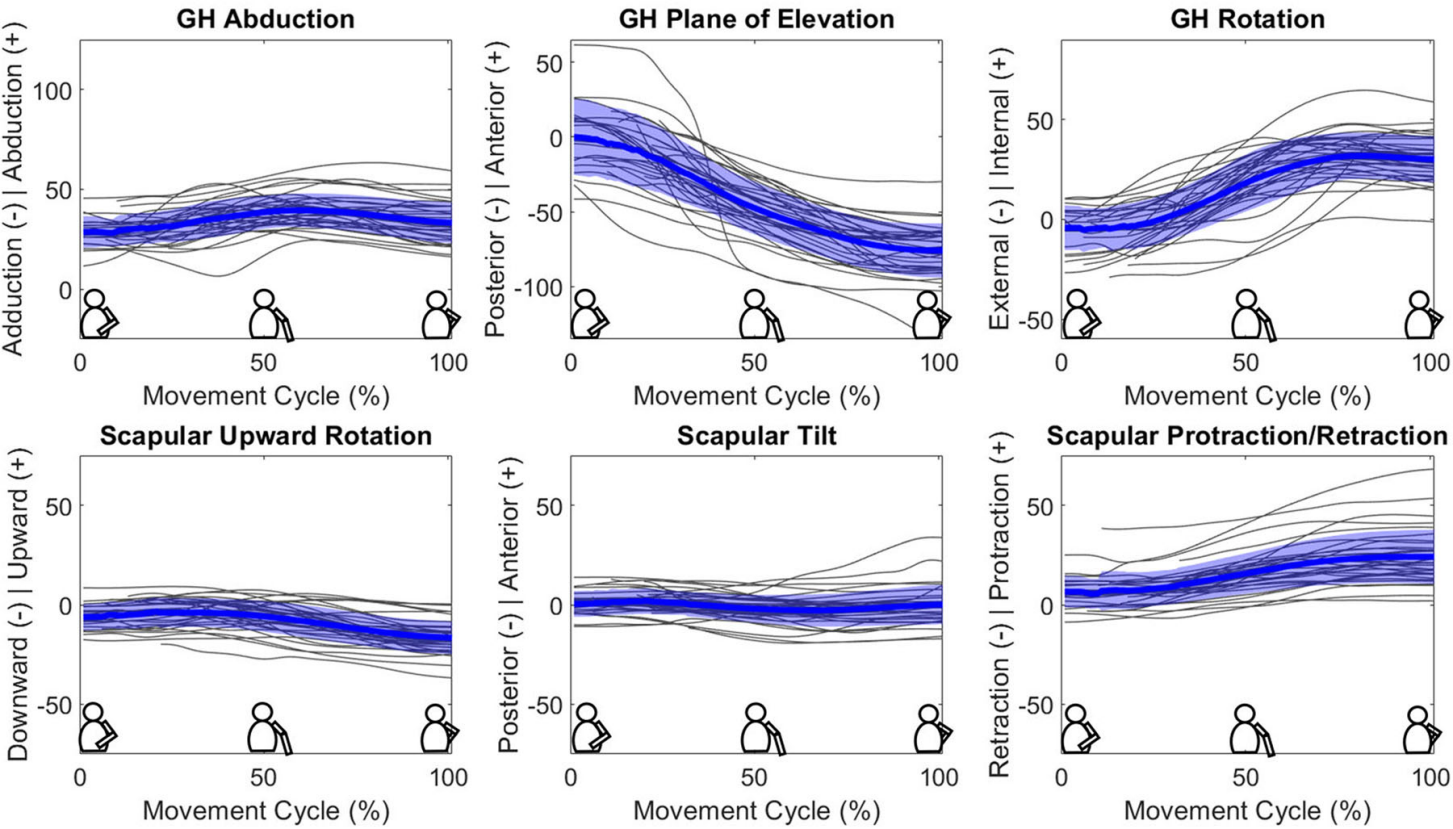
Kinematics of the H2B motion. The thick blue line indicates group average kinematics through the motion, with shaded regions indicating ± 1 standard deviation. Individual kinematics curves for the 35 participants are represented by thin black lines. The Y‐axis is measured in degrees, and the directionality is specified for each motion.

**Figure 5:**
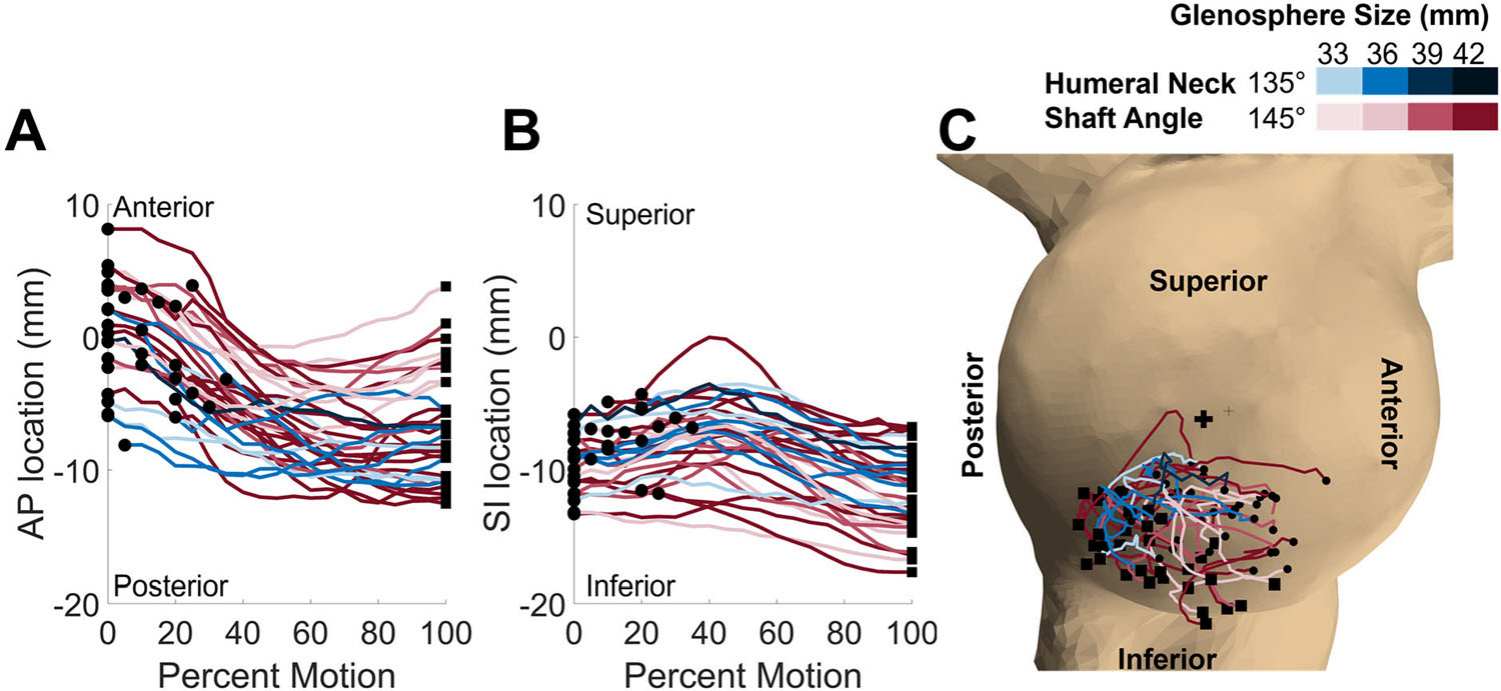
The path of the center of contact of the humeral liner on the glenosphere during the H2B motion. (A) The progression of the center of contact in the anterior‐posterior direction. (B) The progression of the center of contact in the superior‐inferior direction. Increasing values on the y‐axis represent more anterior and superior points, respectively. (C) The path of the center of contact on the glenosphere. Blue lines correspond to patients with 135° humeral neckshaft angle, and red lines correspond to patients with 145° humeral neckshaft angles. Darker shades represent larger glenosphere sizes while lighter shades indicate smaller glenospheres. The motion of each contact path begins at the circle and ends at the square.

The H2B motion was primarily performed via rotations that changed the plane of elevation and GH internal/external rotation. Participants tended to move from an anterior to posterior plane of elevation, and they generally moved from external to internal rotation; in the final 25% of the motion, subjects demonstrated slight ER and adduction while their arms settled. Additionally, scapular protraction contributed to the motion. GH abduction, scapular upward rotation, and scapular tilt were minor contributors to the H2B motion (Figure 4). The center of contact between the humeral liner and the glenosphere generally moved anterior to posterior, and on average, more inferiorly throughout the motion (Figure 5).

**Figure 6:**
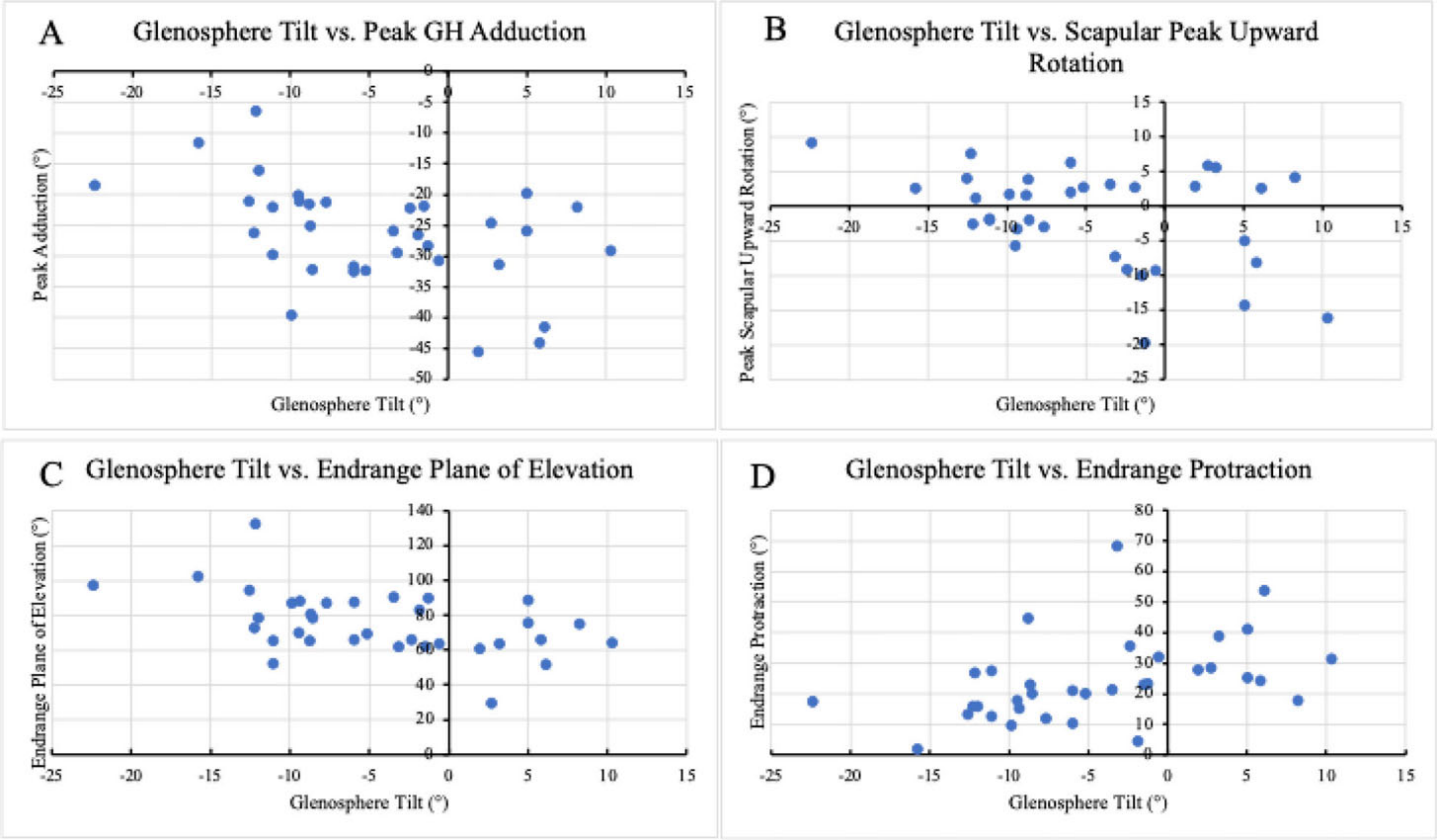
Associations between Glenosphere Tilt and Kinematics. (A) The association between glenosphere tilt and peak glenohumeral adduction. (B) The association between glenosphere tilt and peak upward rotation. (C) The association between glenosphere tilt and endrange plane of elevation. (D) The association between glenosphere tilt and endrange protraction.

**Figure 7:**
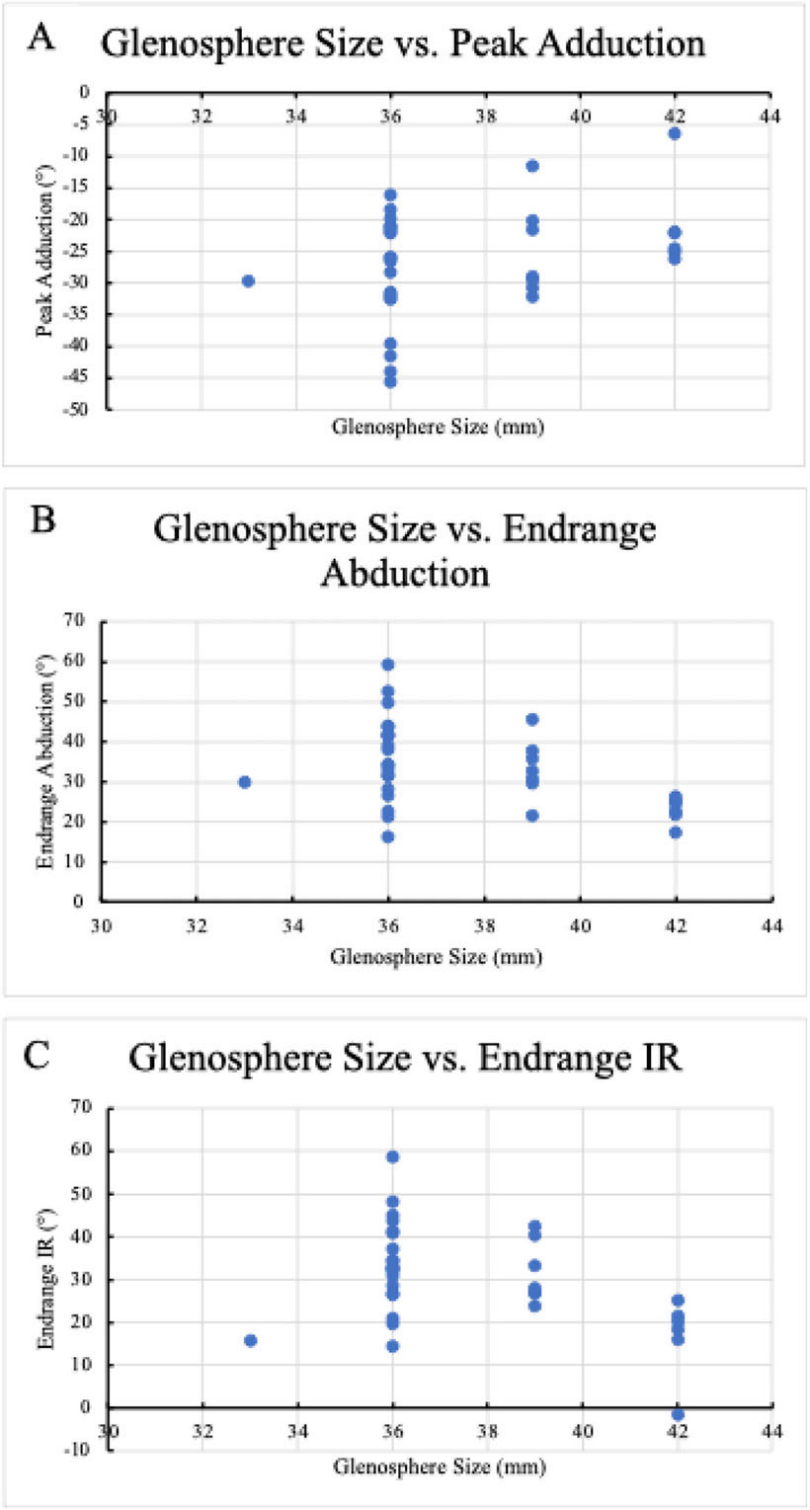
Associations between Glenosphere Size and Kinematics. (A) The association between glenosphere size and peak adduction. (B) The association between glenosphere size and endrange abduction. (C) The association between glenosphere size and endrange IR.

All regression results are included in Table 2 while PRO and strength outcomes are in Table 3. Selected relationships between either glenoid tilt or glenosphere size and kinematics are presented in Figure 6 and Figure 7, respectively.

**Table 2:** Regression analyses results.

	Glenosphere tilt (°)	Glenosphere size (mm)	Glenoid lateralization (mm)	Glenoid eccentricity (mm)	Humeral retroversion (°)	Neck‐Shaft angle (°)
Peak abduction (°)	**0.39 [2.20] (0.04)**	−0.22 [−1.37] (0.18)	−0.19 [−1.13] (0.27)	−0.09 [−0.54] (0.59)	−0.09 [−0.55] (0.59)	0.24 [1.46] (0.15)
Peak internal rotation (°)	−0.07 [−0.27] (0.79)	−1.46 [−1.30] (0.20)	−0.74 [−0.72] (0.48)	−5.01 [−0.83] (0.41)	−0.14 [−0.71] (0.48)	0.56 [0.96] (0.34)
Peak upward rotation (°)	**−0.37 [−2.49] (0.02)**	−0.11 [−0.68] (0.50)	−0.08 [−0.49] (0.63)	−0.09 [−0.54] (0.59)	−0.11 [−0.66] (0.51)	0.17 [1.02] (0.32)
Peak anterior tilt (°) (no significant variables in regression analysis)	−0.32 [−1.67] (0.11)	1.60 [1.99] (0.06)	−0.37 [−0.50] (0.62)	−10.20 [−2.36] (0.03)	−0.08 [−0.53] (0.60)	−0.15 [−0.36] (0.72)
Peak protraction (°)	**0.80 [2.86] (0.01)**	0.24 [1.51] (0.14)	−0.27 [−1.75] (0.09)	0.16 [0.99] (0.33)	−0.09 [−0.57] (0.57)	0.08 [0.48] (0.63)
Peak adduction (°)	**−0.50 [−3.02] (0.00)**	**1.08 [2.12] (0.04)**	0.27 [1.62] (0.12)	−0.10 [−0.53] (0.60)	0.06 [0.36] (0.72)	−0.14 [−0.91] (0.37)
Peak posterior plane of elevation (°)	**−1.07 [−2.85] (0.01)**	−0.05 [−0.30] (0.76)	0.09 [0.56] (0.58)	−0.26 [−1.69] (0.10)	0.10 [0.61] (0.55)	0.06 [0.38] (0.71)
Peak downward rotation (°)	0.10 [0.51] (0.61)	0.37 [0.46] (0.65)	1.10 [1.46] (0.16)	2.50 [0.57] (0.57)	0.10 [0.67] (0.51)	0.26 [0.62] (0.54)
Peak posterior tilt (°)	**0.37 [2.61] (0.01)**	0.12 [0.74] (0.47)	−0.29 [−1.89] (0.07)	0.30 [1.95] (0.06)	0.09 [0.58] (0.57)	0.27 [1.70] (0.10)
Peak retraction (°)	**−0.57 [−3.21] (0.00)**	−0.11 [−0.73] (0.47)	0.24 [1.56] (0.13)	−0.09 [−0.55] (0.59)	0.07 [0.43] (0.67)	−0.13 [−0.84] (0.41)
Abduction endrange (°)	0.27 [1.72] (0.10)	**−1.78 [−2.69] (0.01)**	−0.32 [−1.83] (0.08)	−0.05 [−0.25] (0.81)	−0.02 [−0.09] (0.93)	0.23 [1.44] (0.16)
Plane of elevation endrange (°)	**−1.13 [−3.05] (0.00)**	−0.06 [−0.40] (0.69)	0.10 [0.66] (0.51)	−0.26 [−1.72] (0.10)	0.10 [0.60] (0.55)	0.05 [0.31] (0.76)
Internal/External rotation endrange (°)	0.06 [0.34] (0.74)	**−1.99 [−2.66] (0.01)**	−0.29 [−1.60] (0.12)	−0.08 [−0.39] (0.70)	−0.08 [−0.42] (0.68)	0.23 [1.39] (0.17)
Upward rotation endrange (°)	0.05 [0.24] (0.81)	0.40 [0.46] (0.65)	1.14 [1.44] (0.16)	3.01 [0.66] (0.52)	0.12 [0.80] (0.43)	0.21 [0.48] (0.64)
Tilt endrange (°) (no significant variables in regression analysis)	−0.38 [−1.72] (0.10)	2.09 [2.29] (0.03)	0.01 [0.02] (0.99)	−12.31 [−2.50] (0.02)	−0.09 [−0.58] (0.57)	−0.13 [−0.26] (0.79)
Protraction endrange (°)	**0.79 [2.78] (0.01)**	0.23 [1.44] (0.16)	−0.27 [−1.71] (0.10)	0.14 [0.89] (0.38)	−0.09 [−0.55] (0.59)	0.09 [0.52] (0.61)
Peak posterior contact (mm)	−0.10 [−0.62] (0.54)	**−0.66 [−3.20] (0.00)**	0.06 [0.31] (0.76)	−0.19 [−1.01] (0.32)	−0.27 [−1.59] (0.12)	−0.19 [−1.19] (0.24)
SI contact (mm) (no significant variables in regression analysis)	0.12 [2.15] (0.04)	−0.15 [−0.65] (0.52)	−0.34 [−1.55] (0.13)	1.65 [1.30] (0.20)	−0.05 [−1.15] (0.26)	−0.35 [−2.83] (0.01)
End SI contact (mm)	0.09 [0.59] (0.56)	**0.84 [3.16] (0.00)**	−0.10 [−0.54] (0.59)	0.17 [0.90] (0.37)	0.18 [1.05] (0.30)	0.18 [1.14] (0.26)
End AP contact (mm)	0.19 [1.37] (0.18)	**−0.87 [−4.17] (0.00)**	−0.05 [−0.29] (0.78)	0.06 [0.35] (0.73)	−0.15 [−0.93] (0.36)	−0.21 [−1.46] (0.15)

*Note:* Statistically significant findings using forward selection are in bold font; values are reported as: Beta [T‐statistic] (*p*‐value).

**Table 3:** PROs and strength outcomes [27].

Subject	ASES	DASH	CMS	IR total work (Nm(°)/kg)	IR peak torque (Nm/kg)
1	85.0	30.8	68.1	0.01	0.02
2	61.7	25.0	74.4	0.51	0.38
3	78.3	25.0	73.8	0.27	0.20
4	91.5	30.0	87.0	0.21	0.17
5	95.0	3.3	75.1	0.02	0.12
7	76.8	3.3	75.0	0.18	0.15
8	92.0	17.5	69.2	0.39	0.29
9	83.3	4.2	85.6	0.44	0.30
10	80.3	18.3	78.8	0.23	0.23
11	35.0	35.8	70.0	0.07	0.17
12	89.0	61.7	66.8	0.07	0.11
13	98.3	18.3	69.7	0.02	0.12
14	87.0	1.7	86.0	0.36	0.19
15	91.7	12.5	59.4	0.09	0.10
16	100.0	15.0	68.5	0.15	0.15
17	54.5	5.8	80.2	0.29	0.19
18	93.3	0.0	70.1	0.16	0.11
19	96.7	37.5	67.4	0.13	0.13
20	74.3	2.5	80.1	0.04	0.17
21	51.0	26.7	62.3	0.13	0.12
22	73.3	30.8	68.1	0.23	0.18
23	94.3	34.2	60.2	0.05	0.11
24	92.2	7.5	95.9	0.32	0.18
25	96.7	3.3	84.6	0.05	0.10
26	96.2	6.7	79.8	0.09	0.13
27	89.7	5.0	93.6	0.54	0.41
28	93.3	0.8	54.8	0.06	0.10
29	88.3	7.5	72.7	0.09	0.07
30	64.2	11.7	69.3	0.03	0.20
31	51.5	30.0	57.8	0.00	0.00
32	71.0	14.2	78.4	0.39	0.27
33	85.5	28.3	46.6	0.07	0.12
34	96.7	11.7	88.9	0.45	0.28
35	96.7	9.2	65.4	0.07	0.09
Mean	82.5	16.9	73.0	0.18	0.17
Standard Deviation	16.2	14.1	11.1	0.16	0.09

### 3.3 Glenosphere Tilt and Kinematics

In our analysis of surgical parameters that predicted kinematics, it was found that a less inferior glenosphere tilt predicted the following glenohumeral kinematics: more peak abduction (B = 0.389; *p* = 0.035), less peak adduction (B = −0.499; *p* = 0.005; Figure 6A), less peak posterior plane of elevation (B = −1.068, *p* = 0.008), and less end‐range plane of elevation (B = −1.135; *p* = 0.005; Figure 6C). A less inferior glenosphere tilt also predicted the following scapular kinematics: less peak upward rotation (B = −0.371; *p* = 0.018; Figure 6B), higher maximum protraction (B = 0.797, *p* = 0.007), more protraction at end‐range (B = 0.791; *p* = 0.009; Figure 6D), less peak retraction (B = −0.574; *p* = 0.003), and more peak posterior tilt (B = 0.374; *p* = 0.014).

### 3.4 Glenosphere Size and Kinematics

Glenosphere size also predicted several kinematic parameters. A larger glenosphere predicted more peak adduction (B = 1.076; *p* = 0.042; Figure 7A), less abduction at end‐range (B = −1.778; *p* = 0.011; Figure 7B), less IR at end‐range (B = −1.994; *p* = 0.012; Figure 7C), less peak posterior center of contact (B = −0.658; *p* = 0.003), more anterior center contact at end‐range (B = 0.843; *p* = 0.003), and less superior center of contact at end‐range (B = −0.873; *p* < 0.001).

### 3.5 Glenoid Lateralization, Humeral Retroversion, Neck‐Shaft Angle and Kinematics

No significant associations were found between glenoid lateralization, humeral retroversion, or neck‐shaft angle and kinematics (all *p* > 0.05; Table 2).

### 3.6 Kinematics and Clinical Outcomes

**Figure 8:**
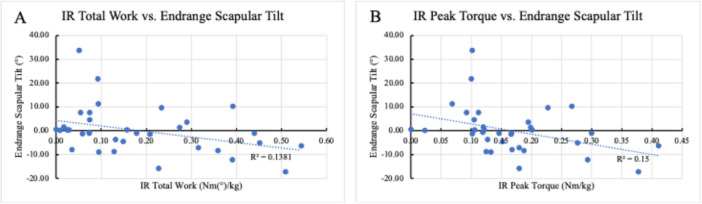
Associations between Peak Strength Measurements and Endrange Scapular Tilt. (A) The association between IR total work and Endrange scapular tilt. (B) The association between IR Peak Torque and Endrange scapular tilt.

**Figure 9:**
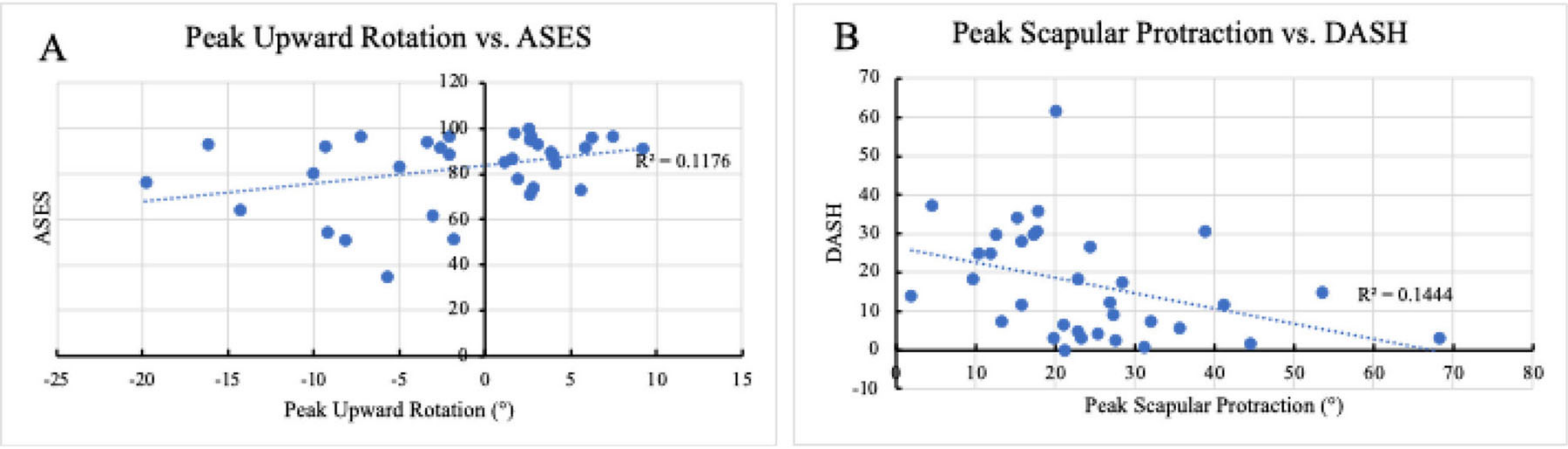
Associations between Kinematics and PROs. (A) The association between peak upward rotation and ASES scores. (B) The association between scapular protraction and DASH scores.

Several associations between kinematics during the hand to back motion and clinical outcomes were observed. There was a fair association between active and passive IR ROM and peak abduction (*r* = 0.384, *p* = 0.025; *r* = 0.402, *p* = 0.019, respectively) and peak IR (*r* = 0.433, *p* = 0.011; *r* = 0.462, *p* = 0.006, respectively) during the H2B motion such that the further a patient could reach up their spine both actively and passively, the more abduction and peak internal rotation in their motion. There was a fair association between passive IR ROM and IR at end‐range (*r* = 0.430; *p* = 0.011). Total work and peak torque in IR had a fair association with posterior scapular tilt (*r* = 0.387, *p* = 0.024; *r* = 0.435; *p* = 0.010, respectively) and anterior scapular tilt at end‐range (*r* = −0.378, *p* = 0.027, Figure 8A; *r* = −0.386, *p* = 0.024, Figure 8B respectively). Strength outcomes were associated with center of contact path values: IR total work (*r* = −0.346; *p* = 0.045) and IR peak torque (*r* = −0.366; *p* = 0.033) both had a fair association with posterior peak center of contact point. There was a fair association between total work in IR and superior contact path (*r* = −0.381; *p* = 0.026). In terms of PROs, there was a fair association between ASES scores and peak upward rotation (*r* = 0.343; *p* = 0.047; Figure 9A). Finally, there was a fair association between DASH scores and peak protraction (Figure 9B) and protraction at end‐range (*r* = −0.389, *p* = 0.023; *r* = −0.380; *p* = 0.027, respectively).

## 4 Discussion

There were a few main findings of this study. First, glenosphere size and tilt predicted in vivo kinematics during H2B after RSA. Additionally, more active and passive IR during clinical exams were associated with more IR and abduction during the H2B movement. Next, scapular kinematics and the location of the center of contact between the humeral liner and glenosphere were associated with strength. Lastly, more upward rotation and scapular protraction were associated with better PROs.

Current beliefs about how implant design and surgical technique affect kinematics after RSA are primarily based upon computer modeling [10, 13]. This study adds much‐needed in vivo data to improve our understanding of these relationships. The kinematic findings from this study support prior in vivo studies that used conventional motion capture and static end‐range radiographs to conclude that abduction, IR, tilt, and upward rotation are kinematics during the H2B movement that are associated with favorable clinical outcomes [17, 21, 28]. RSA may alter the resting position of the scapula postoperatively [29, 30], and the ability to maximize compensatory movement with scapulothoracic motion may be a plausible mechanism for improved outcomes, as seen in Sulkar et al. analysis on RSA kinematics [31]. Similarly, the current study also highlights scapular motion as potentially influential on clinical outcomes. In our analysis, all components of scapular rotation were associated with favorable clinical outcomes, which suggests rehabilitation focused on scapular motion may improve clinical outcomes after RSA. Prior studies have noted more dominant scapular upward rotation in movements such as abduction and flexion RSA [32, 33], while the current study associates greater scapular motion during H2B with favorable clinical outcomes.

Glenosphere tilt predicted numerous glenohumeral and scapular kinematics during the H2B motion, and the associated kinematics, in turn, correlated with clinical outcomes. Specifically, glenosphere tilt predicted glenohumeral ab/adduction and plane of elevation, and scapular rotation and protraction; these kinematics were associated with clinical IR ROM, strength, and more favorable DASH scores. The finding that glenosphere tilt was associated with both more glenohumeral abduction and less scapular upward rotation suggests that glenosphere tilt may influence the ratio between glenohumeral abduction and scapular upward rotation during H2B. However, the ideal tilt remains unclear. Other studies’ findings are contradictory as far as ideal tilt; some report that inferior tilt is beneficial [34] while others do not report any benefit of a specific configuration [35]. Further work is needed to characterize the upper and lower bounds of glenosphere tilt that lead to favorable clinical outcomes after RSA.

Glenosphere size was the other implant parameter that predicted kinematics (mostly contact point locations) that were correlated with clinical outcomes. In this analysis, a larger glenosphere predicted a more anterior and inferior center of contact which was associated with more strength in IR. A potential explanation for this observed relationship is that the larger glenosphere may have increased the muscle moment arms (and therefore increased torque generating capacity of the muscles) which may have shifted the contact center. While conflicting reports exist on the impact of glenosphere size on strength in IR [9, 10, 36], this study may provide a possible mechanism that influences strength‐related findings. However, the association between larger glenosphere size and strength could be confounded by the fact that bigger (and possibly therefore stronger) patients received larger glenosphere implants; however, all strength measures were normalized to bodyweight to partially account for the relationship between size and strength.

Contrary to our hypothesis, humeral retroversion, lateralization, and neck‐shaft angle did not predict kinematics during the H2B motion. There may be other activities of daily living, such as abduction or circumduction, that better reveal the association between surgical technique, implant parameters, and kinematics.

The strengths of this study include a validated system that accurately measures in vivo GH kinematics during functional activities. Accurate in vivo kinematics studies are needed to confirm or refute research findings based upon cadavers or computational models that cannot account for healing or neuromuscular changes that occur after surgery. The experimental design was rigorous in that it included in vivo kinematics, functional outcomes (strength and ROM), and PROs. Multiple repetitions of the H2B movement were analyzed for each participant; this improved the estimate of their typical movement pattern compared to a single repetition [37]. However, no study is without limitations. First, a majority of patients were indicated for RSA due to rotator cuff arthropathy; this particular analysis does not explore outcomes of specific surgical indications. Next, the study was restricted to individuals who had the time, ability, and resources (i.e. transportation) to volunteer for this study study. Notably, these results are limited to the H2B movement; the results may be different during other functional activities. Additionally, kinematics during the H2B movement were not available for the contralateral side, nor have they been reported in healthy adults, so it is not clear how well the RSA procedure restored native or healthy kinematics. Surgical techniques and implant designs were not randomized among patients; in each case the surgeon selected the technique and design believed to be optimal for each patient. Finally, follow‐up had a broad range in our patient population, which may be a potential confounder; however, studies indicate that maximal improvement is achieved at 1‐year follow‐up [38, 39].

In conclusion, the current study results demonstrate that glenosphere size and glenosphere tilt were implant characteristics that predicted GH and scapular kinematics during the H2B motion after RSA, and that more scapular motion during the H2B motion was associated with better PROs. The findings of this study may be used to inform surgical decision‐making and rehabilitation protocols for patients receiving RSA.

## Author Contributions

**Ajinkya A. Rai:** data analysis, writing, revision. **Clarissa M. LeVasseur:** data acquisition, analysis, writing. **Gillian E. Kane:** data acquisition, analysis. **Maria A. Munsch:** data acquisition, analysis. **Christopher J. Como:** data acquisition, analysis. **Alexandra S. Gabrielli:** data acquisition, analysis. **Jonathan D. Hughes:** data analysis, interpretation, writing, revision. **William J. Anderst:** design, analysis, interpretation, revision, approval. **Albert Lin:** design, analysis, interpretation, revision, approval. All authors substantially contributed to research design and execution, manuscript drafting, and approval. All authors have read and approved final manuscript.
